# SARS-CoV-2 N protein induces acute kidney injury in diabetic mice via the Smad3-Ripk3/MLKL necroptosis pathway

**DOI:** 10.1038/s41392-023-01410-x

**Published:** 2023-04-07

**Authors:** Liying Liang, Wenbiao Wang, Junzhe Chen, Wenjing Wu, Xiao-Ru Huang, Biao Wei, Yu Zhong, Ronald C. W. Ma, Xueqing Yu, Hui-Yao Lan

**Affiliations:** 1 Departments of Medicine & Therapeutics, Li Ka Shing Institute of Health Sciences, and Lui Che Woo Institute of Innovative Medicine, The Chinese University of Hong Kong, Hong Kong, China; 2 Department of Clinical Pharmacy, Guangzhou Eighth People’s Hospital, Guangzhou Medical University, Guangzhou, China; 3 The Chinese University of Hong Kong-Guangdong Academy of Sciences/Guangdong Provincial People’s Hospital Joint Research Laboratory on Immunological and Genetic Kidney Diseases, The Chinese University of Hong Kong, Hong Kong, China; 4 Guangdong-Hong Kong Joint Laboratory for Immunological and Genetic Kidney Disease and Medical Research Center, and Departments of Nephrology and Pathology, Guangdong Academy of Medical Science, Guangdong Provincial People’s Hospital, Guangzhou, China; 5 Department of Nephrology, The Third Affiliated hospital, Southern Medical University, Guangzhou, China

**Keywords:** Kidney diseases, Epigenetics

**Dear Editor**,

Kidney is one of major organs attacked by SARS-CoV-2, resulting in acute kidney injury (AKI) in critically ill COVID-19 patients, especially in the elderly and diabetic patients with diabetic kidney disease (DKD).^1,2^ Among SARS-CoV-2 proteins, the N protein can be detectable in damaged tubules in COVID-19 patients with AKI.^2,3^ However, the role and mechanisms of N protein-induced AKI in diabetes remain unclear. By using ultrasound-microbubble-mediated kidney-specific gene transfer technique, we found that SARS-CoV-2 N protein overexpression could dose-and time-dependently induce AKI in non-diabetic mice (db/m), showing tubular necrosis with renal dysfunction including a marked increase in blood urea nitrogen (BUN) and creatinine, which was further increased in diabetic db/db mice at age of 8 weeks and became much more severe in those with older age at 16 and 32 weeks with the development of DKD (Fig. 1a–d, and supplementary Figs. S1 and S2). In addition, kidney overexpression of the N protein also upregulated kidney injury molecule 1 (Kim1), a biomarker for AKI, in db/db mice, especially in those with DKD over 16–32 weeks (Fig. 1e, f, and supplementary Fig. S3a), although there was no difference in expression of renal SARS-CoV-2 N mRNA in db/m and db/db mice at different groups (Fig. 1g). These findings reveal that the SARS-CoV-2 N protein is pathogenic in AKI and is capable of promoting more severe AKI in db/db mice, especially in aged db/db with underlying DKD.

**Fig. 1:**
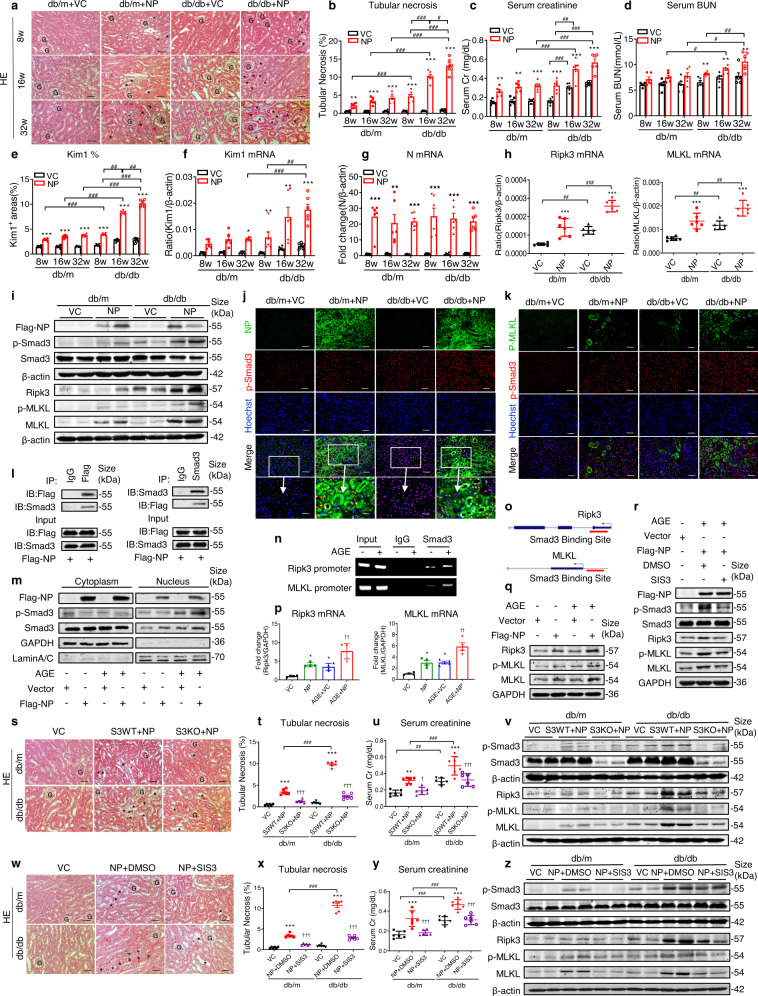
SARS-CoV-2 N Protein induces AKI under diabetic conditions via the Smad3-Ripk3/MLKL necroptosis pathway. **a** Ultrasound-microbubble-mediated kidney-specifically overexpressing SARS-CoV-2 N protein can induce severe tubular necrosis (*) in db/db mice, particularly in those with older age db/db mice (HE-staining, scale bar = 50 μm). **b** Semi-quantitative analysis of tubular necrosis in db/m and db/db mice with or without overexpressing SARS-CoV-2 N protein at the age of 8, 16 and 32 weeks. **c**, **d** Serum levels of creatinine and BUN in db/m and db/db mice with or without overexpressing SARS-CoV-2 N protein at the age of 8, 16, 32 weeks. **e** Quantitative analysis of renal Kim1 protein expression in db/m and db/db mice with or without overexpressing SARS-CoV-2 N protein at the age of 8, 16, 32 weeks. **f**, **g** Quantitative real-time PCR for Kim1 mRNA and SARS-CoV-2 N mRNA expression in db/m and db/db mice with or without overexpressing SARS-CoV-2 N protein at the age of 8, 16, 32 weeks. **h** Quantitative real-time PCR for Ripk3 and MLKL mRNA expression in 16-week-old db/m and db/db mice with or without overexpressing SARS-CoV-2 N protein. **i** Western blot analysis of Flag-NP, p-Smad3, Ripk3, p-MLKL and MLKL expression in the kidneys of 16-week-old db/m and db/db mice with or without overexpressing SARS-CoV-2 N protein. **j** Co-localization between SARS-CoV-2 N protein (green) and p-Smad3 (red) in the AKI kidney (scale bar = 50 μm, and scale bar = 25 μm in amplified images). **k** Co-localization between p-MLKL (green) and p-Smad3 (red) in the AKI kidney (scale bar = 50 μm). **l** Interaction between Flag-SARS-CoV-2 N protein and Smad3 in HK-2 cells by Co-IP. **m** Western blot analysis shows that overexpression of SARS-CoV-2 N protein induces and promotes Smad3 phosphorylation and nuclear translocation in HK-2 cells under high AGE conditions (50 μg/ml for 30 mins). **n**, **o** ChIP assay for detecting the binding of Smad3 to Ripk3 and MLKL promoter region in response to AGE (50 μg/ml). **p** Quantitative real-time PCR for Ripk3 and MLKL mRNA expression in HK-2 cells. **q** Western blot analysis of Ripk3, p-MLKL and MLKL protein expression in HK-2 cells with or without SARS-CoV-2 N protein overexpression and AGE stimulation (50 μg/ml for 6 h). **r** Treatment with SIS3 (10 μM) blocks SARS-CoV-2 N protein-induced activation of Smad3 (p-Smad3) and Ripk3/MLKL signaling in HK-2 cells under high AGE stimulation (50 μg/ml) conditions. **s** Renal pathological changes at the age of 16-week-old Smad3 KO-db/m, Smad3 WT-db/m, Smad3 KO-db/db, and Smad3WT-db/db mice with overexpression of SARS-CoV-2 N protein (HE-staining, scale bar = 50 μm). **t**, **u** Semi-quantitative analysis of tubular necrosis (*) and serum creatinine in Smad3 KO/WT-db/m or Smad3 KO/WT-db/db mice with SARS-CoV-2 N protein overexpression. **v** Western blot analysis of p-Smad3, Ripk3, p-MLKL and MLKL expression in Smad3 KO/WT-db/m or Smad3 KO/WT-db/db mice with overexpressing SARS-CoV-2 N protein. **w** Therapeutic effect of SIS3 (10 mg/kg, daily) on SARS-CoV-2 N-induced renal pathology in db/m and db/db mice (HE-staining, * tubular necrosis, scale bar = 50 μm). **x**, **y** Therapeutic effect of SIS3 on SARS-CoV-2 N-induced tubular necrosis and serum creatinine in db/m and db/db. **z** Western blot analysis shows the inhibitory effect of SIS3 on SARS-CoV-2 N protein-induced p-Smad3, p-MLKL, and expression of Ripk3 and MLKL in the kidney of db/m and db/db mice. The data represents as the mean ± SEM for at least 3 independent experiments in vitro or for groups of 6 mice in vivo. G Glomerulus, S3KO Smad3 knockout mice, S3WT Smad3 wild-type mice, 8w 8 weeks, 16w 16 weeks, 32w 32 weeks; *, necrotic tubules. *P < 0.05, **P < 0.01, ***P < 0.001 vs. VC group; ^#^*P* < 0.05, ^##^*P* < 0.01, ^###^*P* < 0.001 as indicated; ^†^*P* < 0.05, ^††^*P* < 0.01, ^†††^*P* < 0.001 vs. AGE + VC, S3WT + NP, or NP + DMSO group

Mechanistically, consistent with previous reports that the N protein can bind to Smad3,^4,5^ here we also uncover that the N protein can cause AKI in diabetes by inducing tubular cell death via the Smad3-receptor interacting protein kinase 3 (Ripk3)/Mixedlineage kinase domain-like protein (MLKL) necroptosis pathway. Indeed, Smad3 is markedly activated in the diabetic kidney in response to TGF-β1, advanced glycation end products (AGEs), and angiotensin II (Ang II).^6^ Thus, once SARS-CoV-2 N protein is overexpressed, it could bind and promote further Smad3 signaling and Smad3-dependent RIPK3/MLKL necroptosis pathway, resulting in progressive AKI in diabetic mice, particularly in those with DKD (Fig. 1h, i, supplementary Figs. S4 and S5). This was demonstrated by nucleated co-localization of the SARS-CoV-2 N protein and phospho-Smad3 in the AKI kidneys (Fig. 1j). Interestingly, SARS-CoV-2 N protein-induced Smad3 activation induced by overexpress was also associated with increased phosphorylation of MLKL in tubular cells (Fig. 1k). As Ripk3/MLKL-mediated necroptosis is a key mechanism of AKI, it is possible that increased SARS-CoV-2 N protein expression in the kidney may cause tubular necrosis by triggering activation of the Smad3-Ripk3/MLKL necroptosis signaling. This was further confirmed in a human tubular cell line (HK-2) that are overexpressing SARS-CoV-2 N protein in which Co-Immunoprecipitation (Co-IP) detected that the N protein could physically bind Smad3 (Fig. 1l) and thus enhanced Smad3 phosphorylation and nuclear translocation upon AGE stimulation (Fig. 1m, supplementary Fig. S6a, b). Importantly, we also detected that Smad3 was capable of binding to Ripk3 and MLKL promoter region respectively and this physical binding was significantly enriched upon AGE stimulation as shown by chromatin immunosuppression (ChIP) assay (Fig. 1n, o). Thus, when SARS-CoV-2 N protein was overexpressed, it largely enhanced Smad3-Ripk3/MLKL signaling under high AGE condition (Fig. 1p, q, supplementary Fig. S6c), which was blocked by addition of a Smad3 inhibitor SIS3 (Fig. 1r, supplementary Fig. S6d). Furthermore, blockade of the necroptosis pathway with GSK-872, an inhibitor of Ripk3 kinase activity, was also capable of inhibiting Kim1 expression and TGF-β/Smad3 signaling in AGE-stimulated HK-2 cells that overexpressed SARS-CoV-2 N protein (supplementary Fig. S6e–g), revealing a Smad3-Ripk3/MLKL circuit mechanism in SARS-CoV-2 N protein-induced AKI in the diabetic kidney.

To further explore the necessary role of Smad3 in SARS-CoV-2 N-induced AKI, we overexpressed the SARS-CoV-2 N protein in the kidneys of Smad3 KO-db/m, Smad3 WT-db/m, Smad3 KO-db/db, and Smad3 WT-db/db mice at the age of 16 weeks. Strikingly, compared to the Smad3 WT-db/m or Smad3 WT-db/db mice, Smad3 deficiency protected against the SARS-CoV-2 N-induced AKI in Smad3 KO-db/m or Smad3 KO-db/db mice as demonstrated by rare tubular necrosis with normal levels of serum creatinine, BUN, and Kim1 expression (Fig. 1s–u, supplementary Fig. S7). Interestingly, deletion of Smad3 did not alter expression of SARS-CoV-2 N mRNA in the kidney (supplementary Fig. S7a). Moreover, deletion of Smad3 from db/m or db/db mice almost competitively blocked SARS-CoV-2 N-induced Ripk3/MLKL expression and phospho-MLKL level (Fig. 1v, supplementary Fig. S8). Thus, we concluded that Smad3 is necessary for SARS-CoV-2 N-triggered AKI in diabetes via the Ripk3/MLKL-dependent mechanism.

Next, we developed a novel therapy for SARS-CoV-2 N-induced AKI by daily treating diabetic or non-diabetic mice (age of 16 weeks) with a Smad3 inhibitor SIS3 or control DMSO at dosages of 5, 10, or 15 mg/kg body weight intraperitoneally (ip) from the day before the SARS-CoV-2 N gene transfer. Compared to DMSO control, SIS3 treatment dose-dependently attenuated SARS-CoV-2 N protein-caused AKI by markedly inhibiting tubular necrosis, reducing serum creatinine and BUN, and suppressing tubular Kim1 expression in both db/m and db/db mice, with a better therapeutic dose at 10 mg/kg body weight (Fig. 1w–y, supplementary Figs. S9 and S10a–d). However, SIS3 treatment did not influence renal mRNA expression of SARS-CoV-2 N and did not produce systemic toxicity as determined by AST, ALT and LDH assays (supplementary Fig. S10e–g).

As expected, SIS3 treatment also resulted in a marked inhibition of SARS-CoV-2 N-triggered Smad3-Ripk3/MLKL signaling in db/m and db/db mice (Fig. 1z, supplementary Fig. S11), demonstrating that inhibition of Smad3-dependent Ripk3/MLKL necroptosis pathway may be a mechanism through which blockade of Smad3 attenuates SARS-CoV-2 N-induced AKI in diabetes.

In summary, we identified that SARS-CoV-2 N is pathogenic and can cause severe AKI in diabetic mice via the Smad3-Ripk3/MLKL necroptosis pathway, specifically in those with older age and DKD. Targeting this pathway with a Smad3 inhibitor SIS3 can attenuate SARS-CoV-2 N-induced AKI in db/db mice, suggesting SIS3 as a novel therapeutic agent for COVID-19 AKI in diabetic patients (supplementary Fig. S12). However, we also recognized that the impact of this study is limited due to the use of a viral protein rather than live viral infection. In addition, SARS-CoV-2 N protein may trigger multiple cell death pathways to induce AKI as reported here via the Smad3-RIPK3/MLKL necroptosis pathway and the other study under ischemic conditions via the Smad3-p21-dependent apoptosis mechanism.^5^

## Supplementary information

Supplementary materials and Figures

## Data Availability

All data and materials presented in this study are available on request.
