# Supplementary material for: SARS-CoV-2 N protein induces acute kidney injury in diabetic mice via the Smad3-Ripk3/MLKL necroptosis pathway
Source: Signal Transduct Target Ther. 2023 Apr 7;8:147. doi: 10.1038/s41392-023-01410-x (PMC10080522; doi:10.1038/s41392-023-01410-x)
Supplement: Supplementary file 1 — Supplementary materials and Figures [file 41392_2023_1410_MOESM1_ESM.docx]

Supplementary Materials for

SARS-CoV-2 N Protein induces acute kidney injury in diabetic mice via the Smad3-Ripk3/MLKL necroptosis pathway

Liying Liang^1,3,5^, Wenbiao Wang^2,5^, Junzhe Chen^1, 4^, Wenjing Wu^1, 2, 5^, Xiao-Ru Huang^1,2,5^, Biao Wei^1^, Yu Zhong^1^, Ronald CW Ma^1^, Xueqing Yu^2,5*^, Hui-Yao Lan^1,2,5*^.

^1^ Departments of Medicine & Therapeutics, Li Ka Shing Institute of Health Sciences, and Lui Che Woo Institute of Innovative Medicine, The Chinese University of Hong Kong, Hong Kong, China.

^2^ Guangdong-Hong Kong Joint Laboratory for Immunological and Genetic Kidney Disease and Medical Research Center, and Departments of Nephrology and Pathology, Guangdong Academy of Medical Science, Guangdong Provincial People’s Hospital, Guangzhou, China.

^3^ Department of Clinical Pharmacy, Guangzhou Eighth People's Hospital, Guangzhou Medical University, Guangzhou, China.

^4^ Department of Nephrology, The Third Affiliated hospital, Southern Medical University, Guangzhou, China.

^5^The Chinese University of Hong Kong-Guangdong Academy of Sciences/Guangdong Provincial People's Hospital Joint Research Laboratory on Immunological and Genetic Kidney Diseases, The Chinese University of Hong Kong, Hong Kong, China.

Correspondence to: Hui-Yao Lan, E-mail: [hylan@cuhk.edu.hk](mailto:hylan@cuhk.edu.hk);

and Xueqing Yu, E-mail: [yuxueqing@gdph.org.cn](mailto:yuxueqing@gdph.org.cn)

**This PDF file includes:**

Materials and Methods

Supplementary Figure S1 to S12

Materials and Methods

**SARS-CoV-2 N protein expressing plasmid preparation**

Mammalian expression plasmids for pcDNA3.1(+)-Flag-N were constructed and synthesized by GeneScript (Nanjing, China) and the GenBank accession number is MW617760.1. The purification of pcDNA3.1 (+)-Empty-Vector and pcDNA3.1 (+)-Flag-N was according to the manufacturer’s instructions from EndoFree Maxi Plasmid Kit (DP117, TIANGEN BIOTECH CO., LTD, Beijing, China). The primers used in this study are as follow:

Flag-N: F: GCGGATCCATGTCTGATAATGGACCCCA;

R: GCTCTAGATTAGGCCTGAGTTGAGTCAG;

**Generation of transgenic mice**

Smad3 KO (Smad3^-/-)^ mice (C57BL/6) were generated by targeted disruption of the Smad3 gene with homologous (KO) recombination as previously described.^1^ The Smad3^+/-^-db/m mice were generated by cross-breeding with heterozygous Smad3^+/-^ mice to Lepr^+/-^ (db/m) mice (C57BL/6). Double-heterozygous Smad3^+/-^-db/m mice were intercrossed to generate double mutation Smad3 KO-db/db mice and their control littermates including Smad3 WT-db/db mice, Smad3 WT-db/m mice and Smad3 KO-db/m mice. The mouse genotype was identified by extracting and purifying their tail DNA and genotyping in QIAxcel Advanced electrophoresis system. The animal experimentations were approved by the Animal Experimentation Ethics Committee (AEEC) and the Chinese University of Hong Kong.

**Mouse AKI model induced by kidney-specifically transfecting SARS-CoV-2 N-expressing plasmid**

It is well established that db/db mice develop diabetes at 8 weeks of age and DKD at 16 weeks of age, ^2,3^ which becomes more severe with age. Thus, in the present study, db/db mice at the age of 8 weeks were used as the younger age of mice without underlying kidney injury, whereas db/db mice over the age of 16-32 weeks are selected as those with older age and underlying DKD.

To determine the optimal dose and time of SARS-CoV-2 N-induced AKI in normal db/m or db/db mice with or without underlying DKD, db/m or db/db mice at 8, 16, and 32 weeks received ultrasound-microbubble-mediated empty vector or SARS-CoV 2 N-expressing plasmid, respectively. Briefly, mammalian expression plasmids for pcDNA3.1(+)-Flag-N were constructed and synthesized by GeneScript (Nanjing, China) as previously described.^4^ Then the SARS-CoV-2 N protein-expressing plasmid (N) or empty control vector (VC) at 100, 200, 300 µg/mouse was mixed with SonoVue microbubbles (Bracco Diagnostics, Switzerland) at the 1:1 ratio in volume. Total of 400 µL mixture was injected into mice via tail vein, followed immediately by placing the 1.5 cm ultrasound probe (Therasonic 450, Electro Medical Supplies, Wantage, Oxfordshire, England) on the back of mouse opposite the bilateral kidneys and treated with ultrasound at a plus-wave output (2W/cm^2^) for a total of 10 minutes with the 30-second interval to transfect the N gene specifically into the mouse kidneys as previously described.^4,5^ Similarly, to study the role of Smad3 in SARA-CoV-2-induced AKI under diabetic conditions, the same procedure was also used to transfect the SARS-CoV-2 N-expressing plasmid or empty vector to the kidney of Smad3 KO-db/db, Smad3 WT-db/db, Smad3 KO-mice db/m, and Smad3 WT-db/m mice. The animal experimentations were approved by the Animal Experimentation Ethics Committee (AEEC) and the Chinese University of Hong Kong.

**Treatment of SARS-CoV-2 N protein-induced AKI in db/db mice with a Smad3 inhibitor SIS3**

In order to determine an optimal therapeutic dosage of SIS3, db/db mice at the age of 16 weeks were intraperitoneally injected with DMSO (D8418, Sigma-Aldrich Corp, USA) or SIS3 (S7959, Selleck Chemicals, USA) at the dosages of 5 mg/kg, 10 mg/kg or 15 mg/kg body weight daily from the day before SARS-CoV-2 N-expressing plasmid transfer until being sacrificed on day 2.

After determining the optimal therapeutic dosage of SIS3 without systemic toxicity, the db/m and db/db mice at the age of 16 weeks received SARS-CoV-2 N plasmid or control empty vector were treated with DMSO or SIS3 (10 mg/kg) via daily i.p. until being sacrificed on day 2. The animal experimentations were approved by the Animal Experimentation Ethics Committee (AEEC) and the Chinese University of Hong Kong.

**Cell Culture**

The human tubular epithelial (HK-2) cells were purchased from American Type Culture Collection (ATCC) (Manassas, VA, USA). HK-2 cells were cultured with DMEM/F12 medium (Gibco, USA) containing 10% fetal bovine serum (Gibco, USA) and 1% penicillin-streptomycin sulfate (Gibco, ThermoFisher) in the cell incubator with 5% CO_2_ at 37°C. To examine the inhibitory effect of SIS3 on SARS-CoV-2 N protein-induced activation of Smad3-Ripk3/MLKL signaling, HK-2 with overexpressing SARS-CoV-2 N protein was obtained as previously described,^6^ and were stimulated with or without AGE (50μg/ml, ab51995, Abcam, USA) for over the 6-hour period in the presence or absence of 0.1% DMSO or a Smad3 inhibitor SIS3 (10µM, S7959, Selleck Chemicals, USA) 2 hours prior to addition of AGE stimulation.

It is well established that necroptosis plays a role in cell death during AKI.^7-9^ To examine the importance of Ripk3/MLKL necroptosis axis, GSK-872 (1µM, S846502, Selleck Chemicals, USA) was used to inhibit Ripk3 kinase activity as previously descripted.^7,8^ SARS-CoV-2 N-overexpressing HK-2 cells were also stimulated with AGE for 6 and 24 hours in the presence of DMSO or GSK-872 2 hours prior to addition of AGE stimulation. As deletion of Ripk3 and/or MLKL also inhibits the phosphorylation of Smad3 in a mouse model of AKI, ^9^ we also detected the feedback mechanism between Ripk3/MLKL and TGF-β/Smad3 signaling pathways in SARS-CoV-2 N-overexpressing HK-2 cells in the presence or absence of AGE (50μg/ml), GSK-872 (1μM), or TGF-β1 neutralizing antibody (10µg/mL, MAB240, R&D systems, USA ).

**Renal function and histology**

After being anesthetized, mice were sacrificed by drawing blood from heart under anesthesia. Creatinine in serum and urine was measured by the Stanbio Direct Creatinine LiquiColor Test (0430-120, Stanbio Laboratory, USA). The blood urea nitrogen (BUN) detection Kit (C013-2-1，Nanjing Jiancheng Bioengineering Institute, Nanjing, China) was used for detection of serum BUN levels. Hematoxylin and eosin (H&E) staining was performed in 3μm paraffin sections of kidney tissues for detecting tubular necrosis as previously described.^10,11^

**Measurement of alanine aminotransferase (ALT), aspartate aminotransferase (AST) levels and serum lactate dehydrogenase (LDH)**

The aspartate aminotransferase (AST) and alanine aminotransferase (ALT) detection Kits (C010-2, C009-2, Nanjing Jiancheng Bioengineering Institute, Nanjing, China) were used for determination of serum AST and ALT levels. The AST and ALT levels in serum were calculated according to the standard curve and the absorbance of sample at examination wavelength of 510nm.

The lactate dehydrogenase (LDH) detection Kit (A020-2, Nanjing Jiancheng Bioengineering Institute, Nanjing, China) was used for determination of serum LDH levels. The LDH level in serum was calculated according to the standard concentration and the absorbance of blank, sample and standard solution at wavelength of 450nm.

**Immunohistochemistry**

Immunohistochemistry was performed on paraffin-embedded tissue sections (3μm) by a microwave-based antigen retrieval technique.^12^ After incubation with primary antibodies at 4 ℃ overnight, the sections were incubated with anti-rabbit EnVision+ System-HRP Labelled Polymer (K4003, DAKO) at room temperature for 30min-1h, and then color was developed by diaminobezidine (DAB) (D5905, Sigma-Aldrich Corp, USA). The nuclei were counterstained with Hematoxylin (H-3404, Burlingame, USA) if necessary. The Immunohistochemistry staining was detected and photographed by LEICA DM 2500 Automated Upright Microscope System. The primary antibodies used in this study were as follows: Rabbit antibodies against Kim1 (bs-2713R, Bioss), Ripk3 (ab62344, Abcam), p-Smad3 (38202, ROCKLAND).

**Immunofluorescence**

The paraffin-embedded kidney sections (3μm) were used for two-color immunofluorescence by incubating with the rabbit antibodies against SARS-CoV-2 N protein (ab273167, Abcam), MLKL (pS345) (ab196436, Abcam) and p-Smad3 (38202, ROCKLAND) respectively overnight. After washing, the sections were then incubated with anti-rabbit EnVision+ System-HRP Labelled Polymer (K4003, DAKO) at room temperature for 30min-1h, and then fluorescence was developed by Alexa Fluor ^TM^ 488 Tyramide Reagent (B40953, Invitrogen) or Alex Fluor ^TM^ 568 Tyramide Reagent (B40956, Invitrogen). The nuclei were counterstained with Hoechst 33342 (H1399, Invitrogen).

The Frozen kidney sections (6μm) were fixed in acetone and block with 5% BSA (prepared with PBS) for 30 minutes at room temperature and then incubated with rabbit antibodies against p-MLKL (ab196436, Abcam) and MLKL (AP14272B, ABGENT) overnight. After being washed with 1X PBS, the sections were incubated with RHO-Goat anti-Rabbit IgG Secondary antibody (AP187R, Chemicon) in dark at room temperature for 1h. The nuclei were counterstained with Hoechst 33342 (H1399, Invitrogen) and the sections were sealed with Fluorescent Mounting Medium (S3023, Dako). The immunofluorescence staining was detected and photographed by LEICA DM 6000B Automated Upright Microscope System.

**Nuclear and Cytoplasmic Extraction**

The extraction of nuclear and cytoplasmic protein fractions was according to the manufacturer’s protocol from NE-PER Nuclear and Cytoplasmic Extraction Kit (78835, Thermo scientific, USA) and the protein fractions were further analyzed by western blotting.

**Western blot analysis**

After being extracted from cells or tissues by RIPA lysis buffer (P0013B, Beyotime, Shanghai, China), the proteins were separated by sodium dodecylsulfate-polyacrylamide gel electrophoresis (SDS-PAGE) gel and transferred to a Nitrocellulose Transfer Membrane (66485, Pall Corporation, Mexico). Afterwards, the membranes were blocked for 1 h at room temperature with 5 % skim milk (dissolved in TBST) and incubated with the indicated primary antibodies at 4 ℃ overnight, followed by incubation with DyLight™ 800 conjugated anti-rabbit or mouse IgG (H&L) antibody (611-145-002, 610-145-002, ROCKLAND) for 1 h at about 25 ℃. The near-infrared fluorescent signals on membranes were detected by the LI-COR Odyssey Infrared Imaging System (San Diego, CA, UAS) then analyzed quantitatively by the Image J software (NIH, Bethesda, MD, USA). The primary antibodies used in this study were as follows: rabbit antibodies against p-Smad3 (9520S, Cell Signaling Technology), Smad3 (PA5-32588, Invitrogen), Ripk3 (ab62344, Abcam), p-MLKL (ab196436, ab187091, Abcam), MLKL (AP14272B, ABGENT; ab184718, Abcam) and mouse antibodies against Flag M2 (F1804, Sigma-Aldrich), GAPDH (3315725, Millipore Corp.), β-actin (SC-69879, Santa Cruz), LaminA/C (4777, Cell Signaling Technology)，Kim1(ab78494, Abcam).

**Real-time PCR assays**

Total RNA from mice kidney tissue and HK-2 cells was extracted with TRIzol reagent (TR118, Molecular Research Center) following the manufacturer’s instructions. The reverse transcription was performed by CFX96 Touch^TM^ Real-Time PCR System (BIO-RAD, USA) and real-time quantitative-PCR was performed with iQ^TM^ SYBR Green Supermix (1725121, Bio-Rad, USA) by QuantStudio^TM^ 7 Flex Real-Time PCR System (Invitrogen, USA). The primers used in this study were as follows: SARS-CoV-2 N: forward 5’-CCGCATTACGTTTGGTGGAC-3’, reverse 5’- CCATGTTGAGTGAGAGCGGT-3’; Human-Ripk3: forward 5’-CATAGGAAGTGGGGCTACGAT-3’, reverse 5’-AATTCGTTATCCAGACTTGCCAT-3’; Human-MLKL: forward 5’-AGGAGGCTAATGGGGAGATAGA-3’, reverse 5’-TGGCTTGCTGTTAGAAACCTG-3’; Ms-Ripk3: forward 5’-TCTGTCAAGTTATGGCCTACTGG-3’, reverse 5’- GGAACACGACTCCGAACCC-3’; Ms-MLKL: forward 5’-AATTGTACTCTGGGAAATTGCCA-3’, reverse 5’- TCTCCAAGATTCCGTCCACAG-3’; Ms-Kim1: forward 5’-CAGGGAAGCCGCAGAAAA-3’, reverse 5’- GAGACACGGAAGGCAACCAC-3’; Ms-β-actin: forward 5’-GTGACGTTGACATTCGTAAAGA-3’, reverse 5’- GCCGGACTCATCGTACTCC-3’.

**Co-Immunoprecipitation assays**

The Pierce Co-Immunoprecipitation (Co-IP) Kit (26149, Thermo Scientific, USA) was used for immunoprecipitation assays. Briefly, after HK-2 cells being transfected with Flag-N plasmid, cell lysates were prepared and immunoprecipitated following the manufacturer’s instructions. The antibodies used in this study were as follows: anti-IgG antibody (2729P, Cell Signaling Technology), anti-Flag antibody (F3165, Sigma) and anti-Smad3 antibody (511500, Invitrogen).

**ChIP assays**

HK-2 cells were treated with or without AGE (50μg/ml, ab51995, Abcam, USA) for 2 h. The ChIP assays was performed by a Simple ChIP Enzymatic Chromatin IP Kit (Cell Signaling 9003) according to the manufacturer’s instructions. The antibody against Smad3 (1:50) (9523, Cell Signaling) and IgG as control (2729P, Cell Signaling) were used for immunoprecipitation and then PCR was performed. Primers that targeting the Smad3 binding site on human Ripk3 and MLKL promoter regions were as follows: Smad3-hRipk3 primer: forward 5’-TGAATGTCGGA

GGCTGCGATC-3’, reverse 5’-AGACTGACCCCTGCACAGACA-3’; Smad3-hMLKL primer: forward 5’-CGGCCCCTCCCTTCCTTCCTC-3’, reverse 5’-CCCTCCCCACCCCGCCAATAT-3’.

**Statistical analyses**

All data represents as mean ± standard error (mean ± SEM). In statistical analysis, t-test was used for comparing the data between two groups, and one-way ANOVA analysis followed by Tukey post-test was used for comparison from multiple groups via Graphpad prism statistical software. P < 0.05 was statistically significant.

**Reference**

1 Yang, X. *et al.* Targeted disruption of SMAD3 results in impaired mucosal immunity and diminished T cell responsiveness to TGF-beta. *EMBO J*. **18**, 1280-1291, (1999).

2 Xu, B. H. *et al.* Deletion of Smad3 prevents renal fibrosis and inflammation in type 2 diabetic nephropathy. *Metabolism*. **103**, 154013, (2020).

3 Sharma, K., McCue, P. & Dunn, S. R. Diabetic kidney disease in the db/db mouse. *Am J Physiol Renal Physiol*. **284**, F1138-1144, (2003).

4 Zhang, Y. Y. *et al.* LRNA9884, a Novel Smad3-Dependent Long Noncoding RNA, Promotes Diabetic Kidney Injury in db/db Mice via Enhancing MCP-1-Dependent Renal Inflammation. *Diabetes*. **68**, 1485-1498, (2019).

5 Feng, M. *et al.* TGF-beta Mediates Renal Fibrosis via the Smad3-Erbb4-IR Long Noncoding RNA Axis. *Mol Ther*. **26**, 148-161, (2018).

6 Wang, W. *et al.* SARS-CoV-2 N Protein Induces Acute Kidney Injury via Smad3-Dependent G1 Cell Cycle Arrest Mechanism. *Adv Sci (Weinh)*. **9**, e2103248, (2022).

7 Mandal, P. *et al.* RIP3 induces apoptosis independent of pronecrotic kinase activity. *Mol Cell*. **56**, 481-495, (2014).

8 Li, S. *et al.* SARS-CoV-2 Z-RNA activates the ZBP1-RIPK3 pathway to promote virus-induced inflammatory responses. *Cell Res*, 1-14, (2023).

9 Chen, H. *et al.* RIPK3-MLKL-mediated necroinflammation contributes to AKI progression to CKD. *Cell Death Dis*. **9**, 878, (2018).

10 Fu, S. *et al.* Smad7 protects against acute kidney injury by rescuing tubular epithelial cells from the G1 cell cycle arrest. *Clin Sci (Lond)*. **131**, 1955-1969, (2017).

11 Lai, W. *et al.* C-reactive protein promotes acute kidney injury via Smad3-dependent inhibition of CDK2/cyclin E. *Kidney Int*. **90**, 610-626, (2016).

12 Lan, H. Y., Mu, W., Nikolic-Paterson, D. J. & Atkins, R. C. A novel, simple, reliable, and sensitive method for multiple immunoenzyme staining: use of microwave oven heating to block antibody crossreactivity and retrieve antigens. *J Histochem Cytochem*. **43**, 97-102, (1995).

**Supplementary Figures and Figure legends**


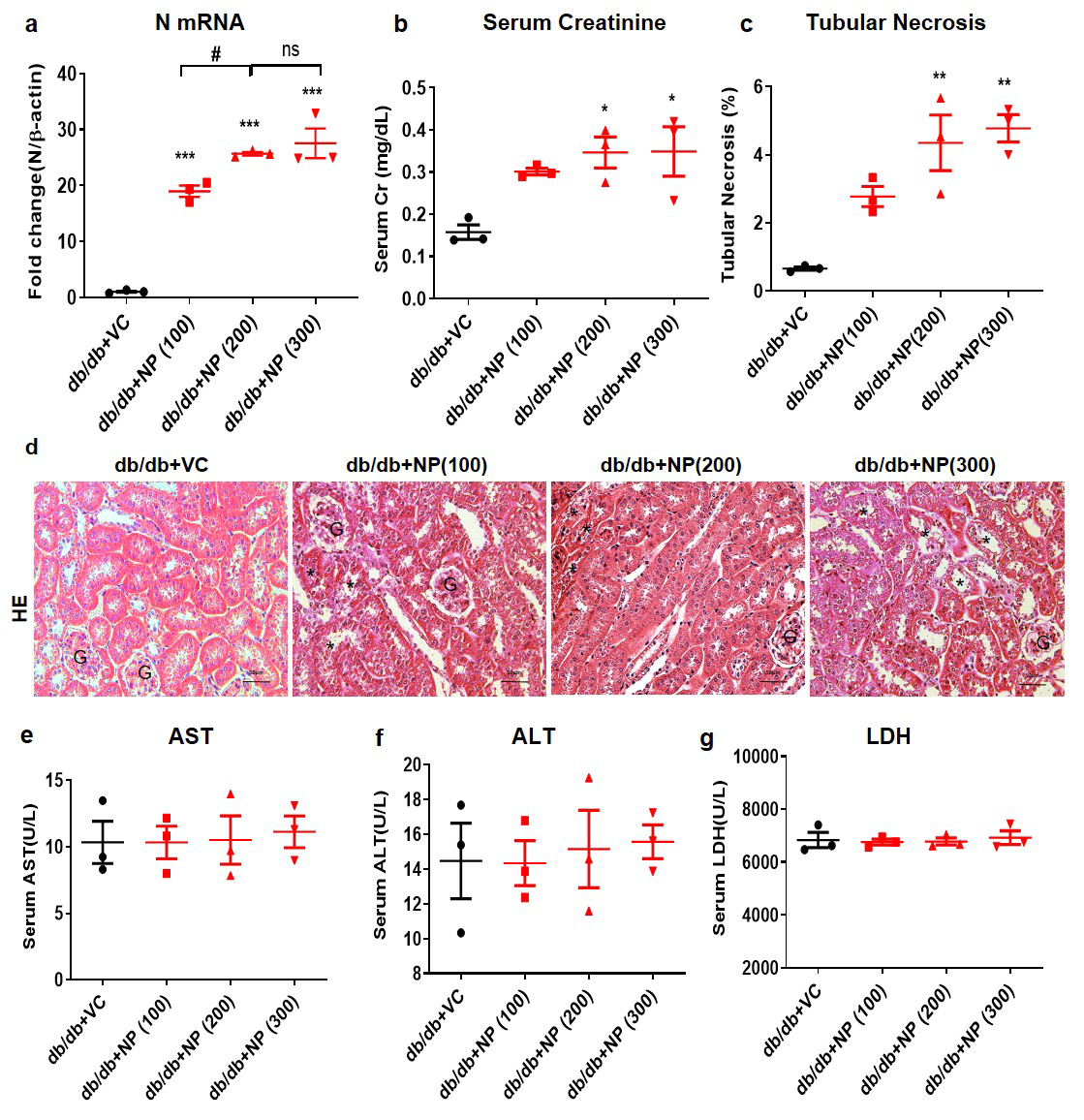


**Supplementary Figure S1. Ultrasound-microbubble-mediated kidney-specifically overexpressing SARS-CoV-2 N mRNA dose-dependently induces AKI in 8-week-old db/db mice without detectable systemic toxicity. a.** SARS-CoV-2 N mRNA expression; **b.** Serum levels of creatinine; **c.** Semi-quantitative analysis of tubular necrosis (*); **d.** H&E-staining; **e-g.** Serum levels of AST, ALT and LDH. Note that kidney-specifically overexpressing SARS-CoV-2 N protein (NP) dose-dependently induces AKI by causing tubular necrosis and elevated serum creatinine, with an optimal dose at 200 μg/mouse without systemic toxicity. Each dot represents one mouse and data are the mean ± SEM per group. *P < 0.05, **P < 0.01, *** p<0.001 vs. db/db+vector control (VC) group; ^#^P < 0.05 as indicated. G, glomerulus; Scale bar=50 μm.


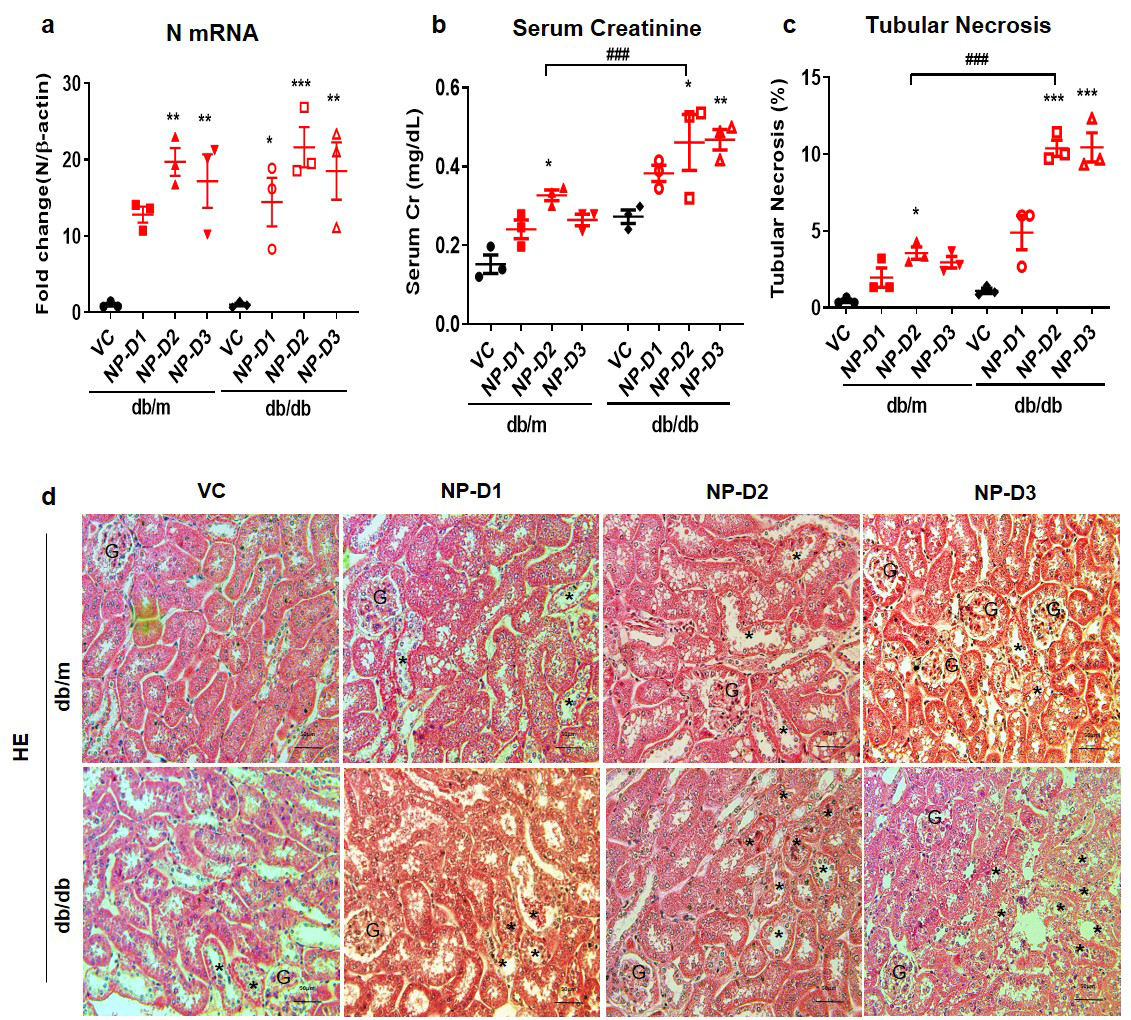


**Supplementary Figure S2. Ultrasound-microbubble-mediated kidney-specifically overexpressing SARS-CoV-2 N mRNA time-dependently induces AKI in 16-week-old db/m and db/db mice, peaking at day 2. a.** SARS-CoV-2 N mRNA expression; **b.** Serum levels of creatinine; **c.** Semi-quantitative analysis of tubular necrosis (*); **d.** H&E-staining. Note that kidney-specifically overexpressing SARS-CoV-2 N mRNA results in increased serum levels of creatinine and tubular necrosis in db/m and db/db mice in a time-dependent manner, peaking at day 2. Each dot represents one mouse and data are the mean ± SEM per group. NP, SARS-CoV-2 N protein, VC, empty vector control. *P < 0.05, **P < 0.01, *** p<0.001 vs. VC group; ^###^P < 0.001 as indicated. G, glomerulus; Scale bar=50 μm.


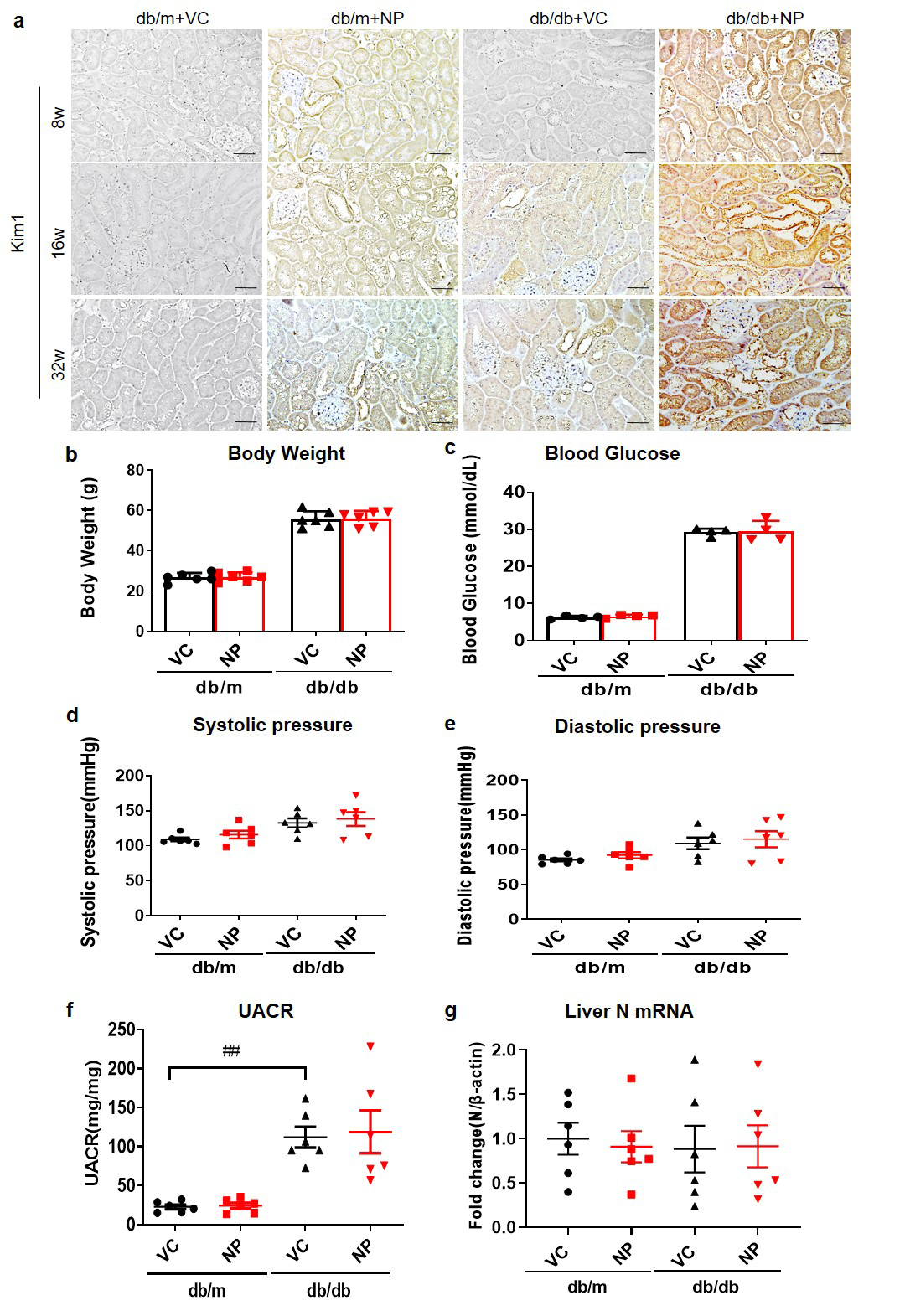


**Supplementary Figure S3. Kidney-specifically overexpressing SARS-CoV-2 N protein (NP) induces AKI without influencing the primary diabetic disease in db/db mice. a.** Immunohistochemistry for renal Kim1 expression in db/m and db/db mice at 8, 16 and 32 weeks (w); **b.** Body weight in 16-week-old mice; **c.** Blood glucose; **d.** Systolic pressure; **e.** Diastolic pressure; **f.** Urinary albumin-to-creatinine ratio (UACR); **g.** qPCR for N mRNA in mouse livers. Note that kidney-specifically overexpressing SARS-CoV-2 N protein (NP) results in upregulation of Kim1 without altering the body weight, blood glucose, blood pressure, and UACR in db/db mice, although db/db mice at the age of 16 weeks develop DKD. VC, empty vector control. Each dot represents one mouse and data are the mean ± SEM for groups of 6 mice.


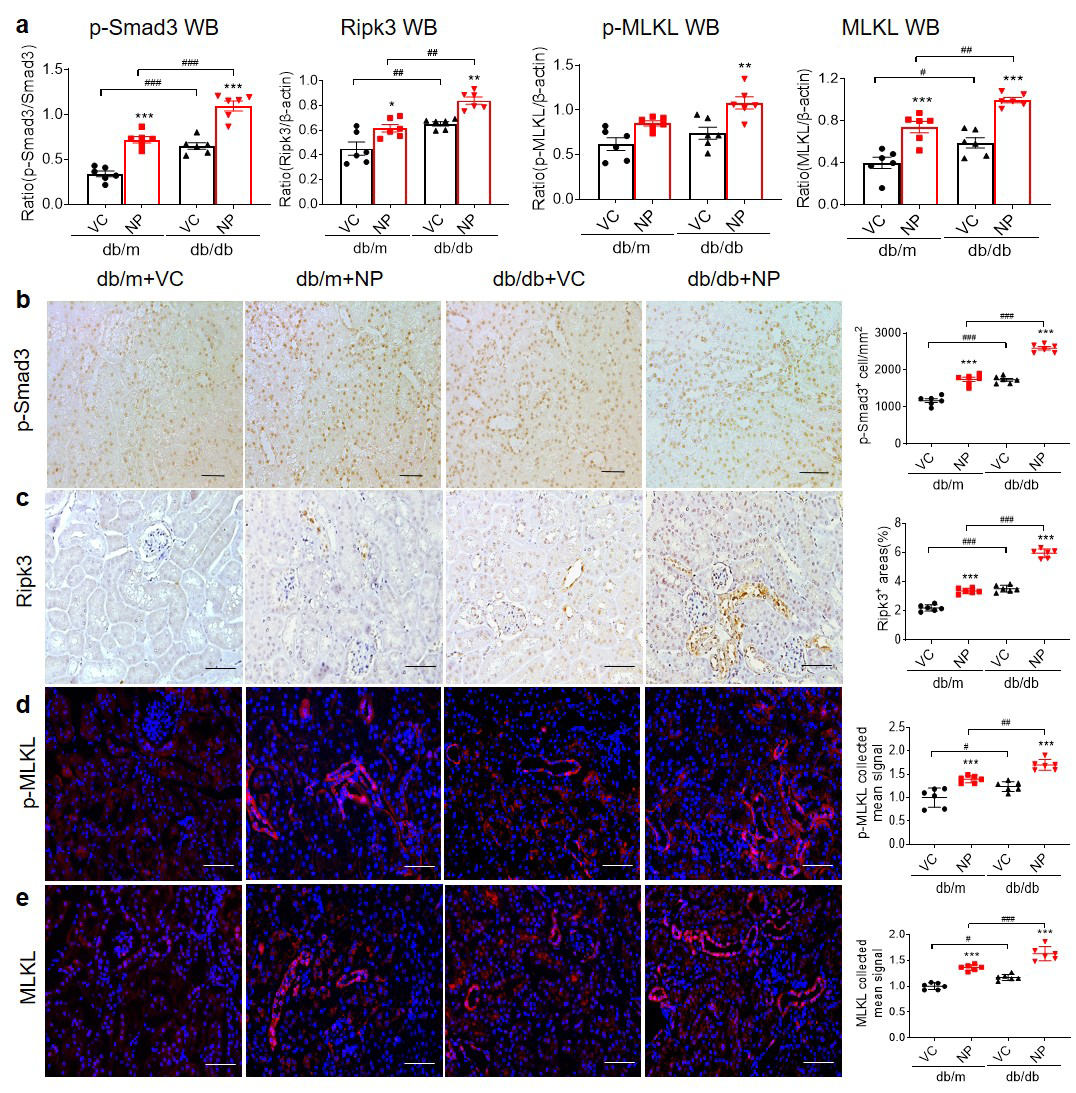


**Supplementary Figure S4. Kidney-specifically overexpressing SARS-CoV-2 N protein (NP) induces activation of Smad3 and Ripk3-MLKL necroptosis pathway in db/m, which is largely enhanced in db/db mice** **at the age of 16 weeks.** **a.** Quantitative analysis of p-Smad3, Ripk3, p-MLKL and MLKL expression by western blotting; **b, c.** Immunohistochemistry and semi-quantitative analysis for Smad3 activation (p-Smad3) and Ripk3 expression; **d, e.** Immunofluorescence and semi-quantitative analysis for p-MLKL and MLKL levels (red). Each dot represents one mouse, and the data represents as the mean ± SEM for groups of 6 mice. VC, empty vector control. **P* < 0.05, ***P* < 0.01, ****P* < 0.001 vs. VC group, ^#^*P* < 0.05, ^##^*P* < 0.01, ^###^*P* < 0.001 as indicated. Scale bar=50 μm.


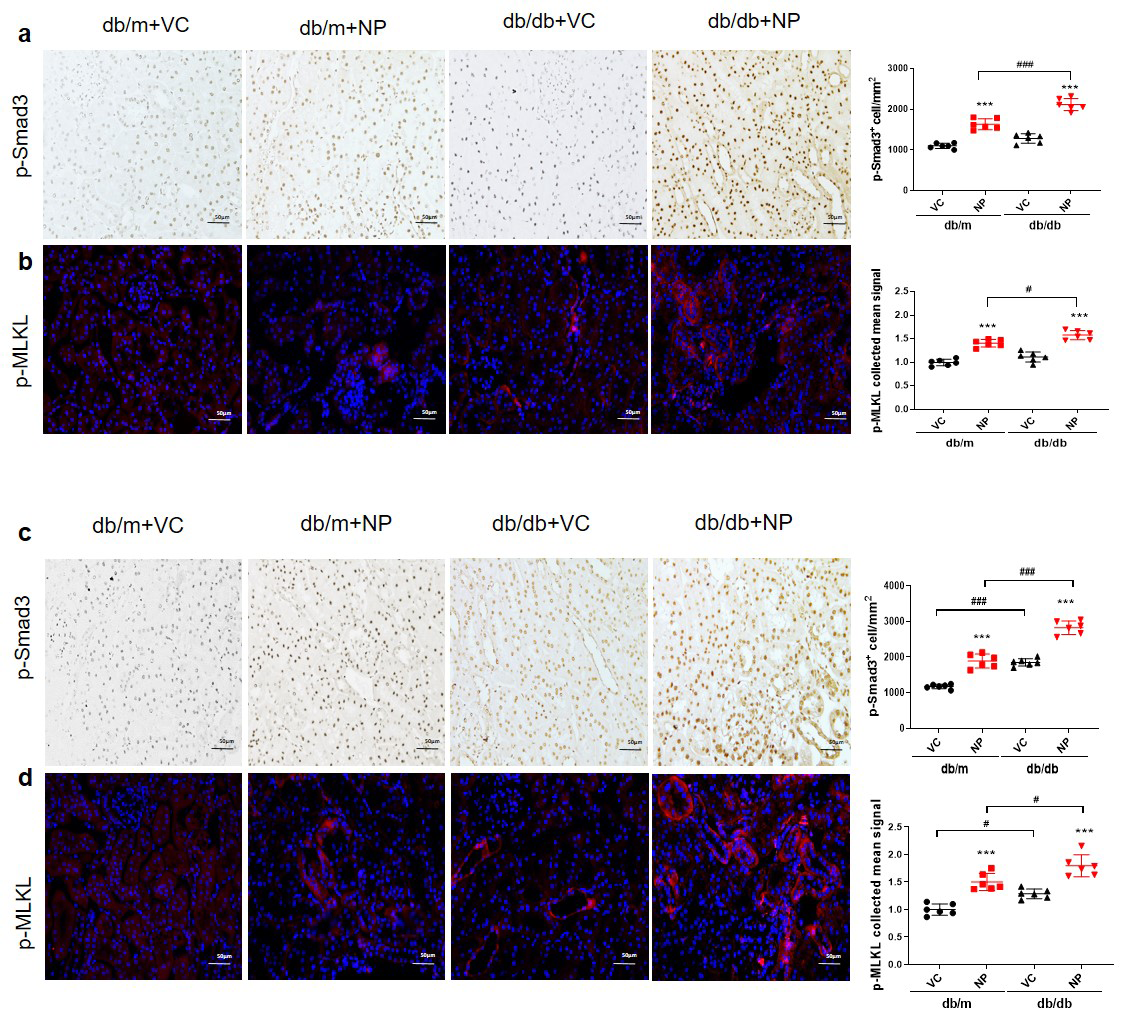


**Supplementary Figure S5. Kidney-specifically overexpressing SARS-CoV-2 N protein increases Smad3 and MLKL phosphorylation in 8- and 32-week-old db/m and db/db mice. a, b.** Immunohistochemistry and semi-quantitative analysis of p-Smad3 and p-MLKL in 8-week-old db/m and db/db mice with or without overexpression of SARS-CoV-2 N protein (NP); **c** Immunohistochemistry and semi-quantitative analysis of p-Smad3 and p-MLKL in 32-week-old db/m/db/db mice with or without overexpression of SARS-CoV-2 N protein (NP). Each dot represents one mouse and data are the mean ± SEM for groups of 6 mice. *** p<0.001 vs. VC group, ^#^*P* < 0.05, ^###^*P* < 0.001 as indicated. Scale bar=50 μm.


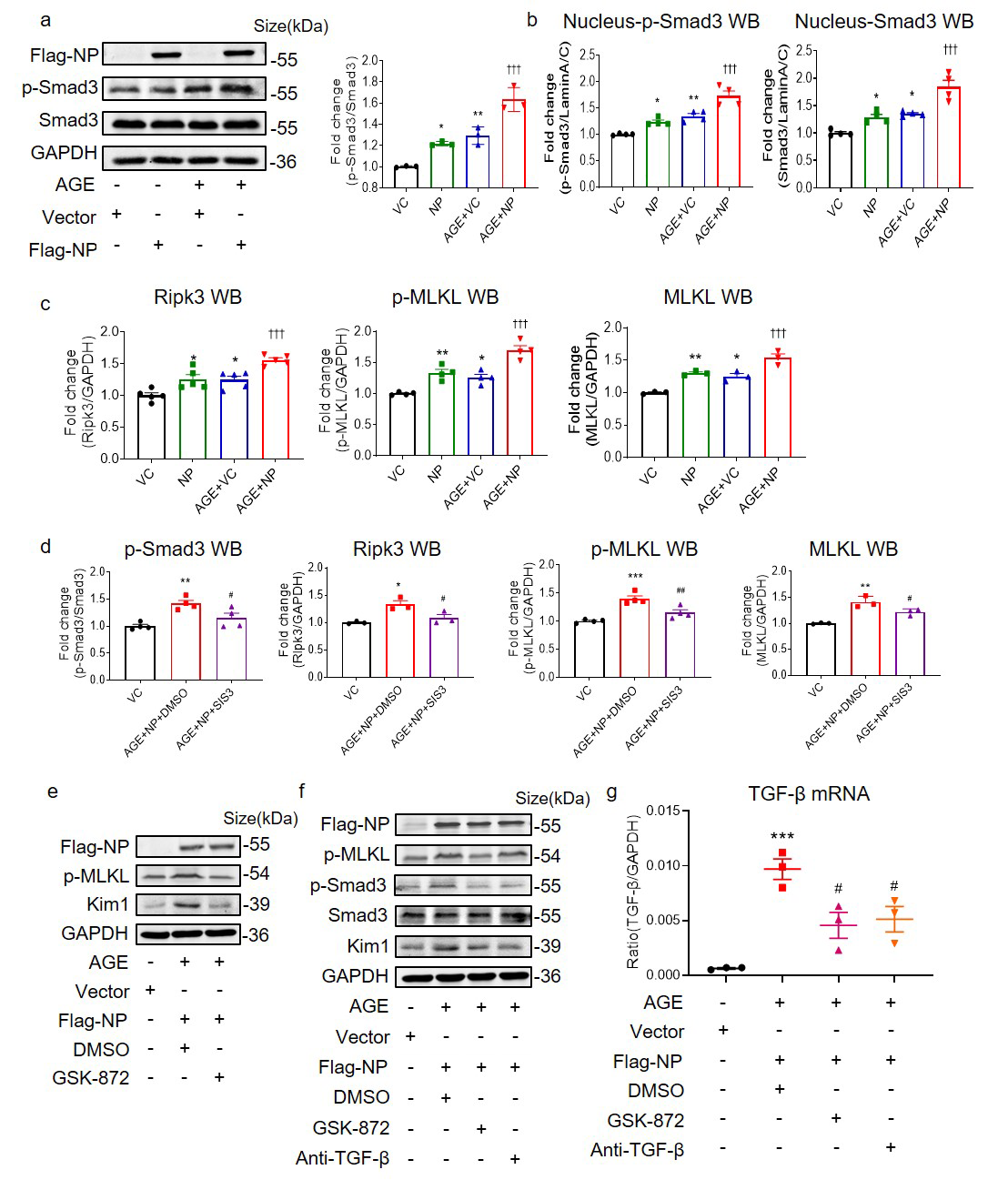


**Supplementary Figure S6. Kidney-specifically overexpressing SARS-CoV-2 N protein promotes Smad3-dependent Ripk3/MLKL necroptosis pathway in HK-2 cells under high AGE conditions. a, b.** Western blot analysis shows that overexpression of SARS-CoV-2 N protein (NP) induces and promotes Smad3 phosphorylation (p-Smad3) and nuclear translocation in HK-2 cells under high AGE conditions (50μg/ml for 30 mins); **c.** Quantitative analysis of Ripk3, p-MLKL and MLKL protein expression in HK-2 cells with or without SARS-CoV-2 N protein overexpression and AGE stimulation (50μg/ml for 6h) by western blot analysis; **d.** Treatment with SIS3 (10μM) blocks SARS-CoV-2 N protein-induced activation of Smad3 (p-Smad3) and Ripk3/MLKL signaling in HK-2 cells under high AGE stimulation (50μg/ml) conditions by western blot analysis; **e.** An inhibitor of Ripk3 kinase activity, GSK-872 (1μM), blocks SARS-CoV-2 N protein-induced Kim1 expression in HK-2 cells under high AGE stimulation (50μg/ml) conditions for 6h; **f, g**. Both GSK-872 and TGF-β neutralizing antibody (10μg/ml) are able to in turn inhibit TGF-β/Smad3 signaling in AGE-stimulated SARS-CoV-2 N-overexpressing HK-2 cells at mRNA levels (6h) and protein levels (24h)**.** The data represents as the mean ± SEM for at least 3 independent experiments. NP, SARS-CoV-2 N protein, VC, empty vector control. **P* < 0.05, ***P* < 0.01, ****P* < 0.001 vs. VC group; ^†^*P* < 0.05, ^††^*P* < 0.01, ^†††^*P* < 0.001 vs. AGE+VC group; ^#^*P* < 0.001 vs. AGE+NP+DMSO group.


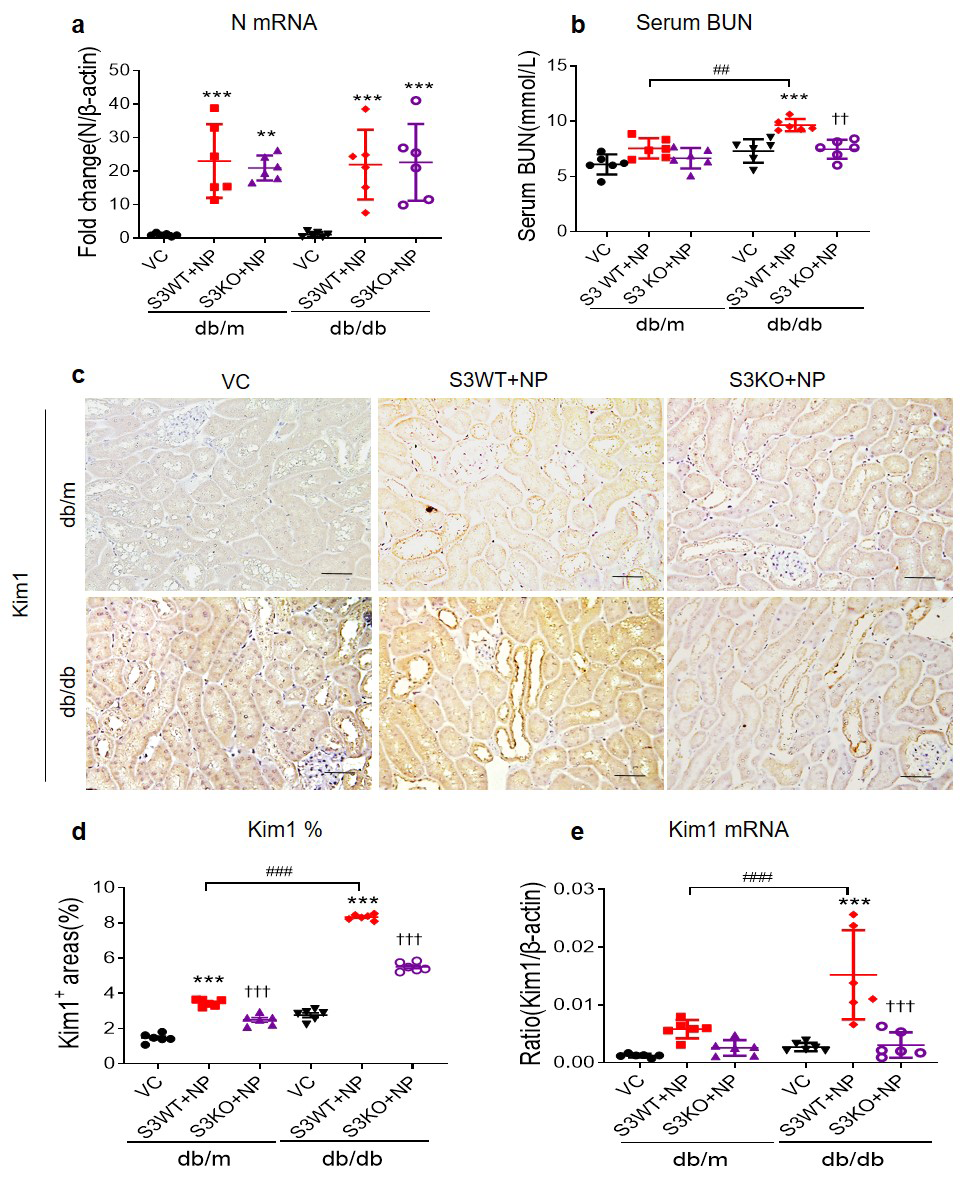


**Supplementary Figure S7. Deletion of Smad3 protects against SARS-CoV-2 N protein-induced AKI in db/m and db/db mice. a.** Quantitative real-time PCR for SARS-CoV-2 N mRNA expression; **b.** Serum levels of BUN; **c, d.** Immunohistochemistry and quantitative analysis of Kim1 expression; **e.** Quantitative real-time PCR for Kim1 mRNA expression in the kidney of db/m and db/db mice at the age of 16 weeks. Note that compared to db/m mice, overexpression of SARS-CoV-2 N protein enhances AKI by increasing tubular necrosis, serum levels of creatinine and BUN, and Kim1 expression in db/db mice at the age of 16 weeks, which is blocked by deleting Smad3. Each dot represents one mouse, and the data represents as the mean ± SEM for groups of 6 mice. S3WT, Smad3 wild-wild-type mice; S3KO, Smad3 knockout mice. VC, empty vector control, NP, SARS-CoV-2 N protein. ***P* < 0.01, ****P* < 0.001 vs. VC group; ^†^*P* < 0.05, ^††^*P* < 0.01, ^†††^*P* < 0.001 vs. S3WT+NP group; ^##^*P* < 0.01, ^###^*P* < 0.001 as indicated. Scale bar=50 μm.


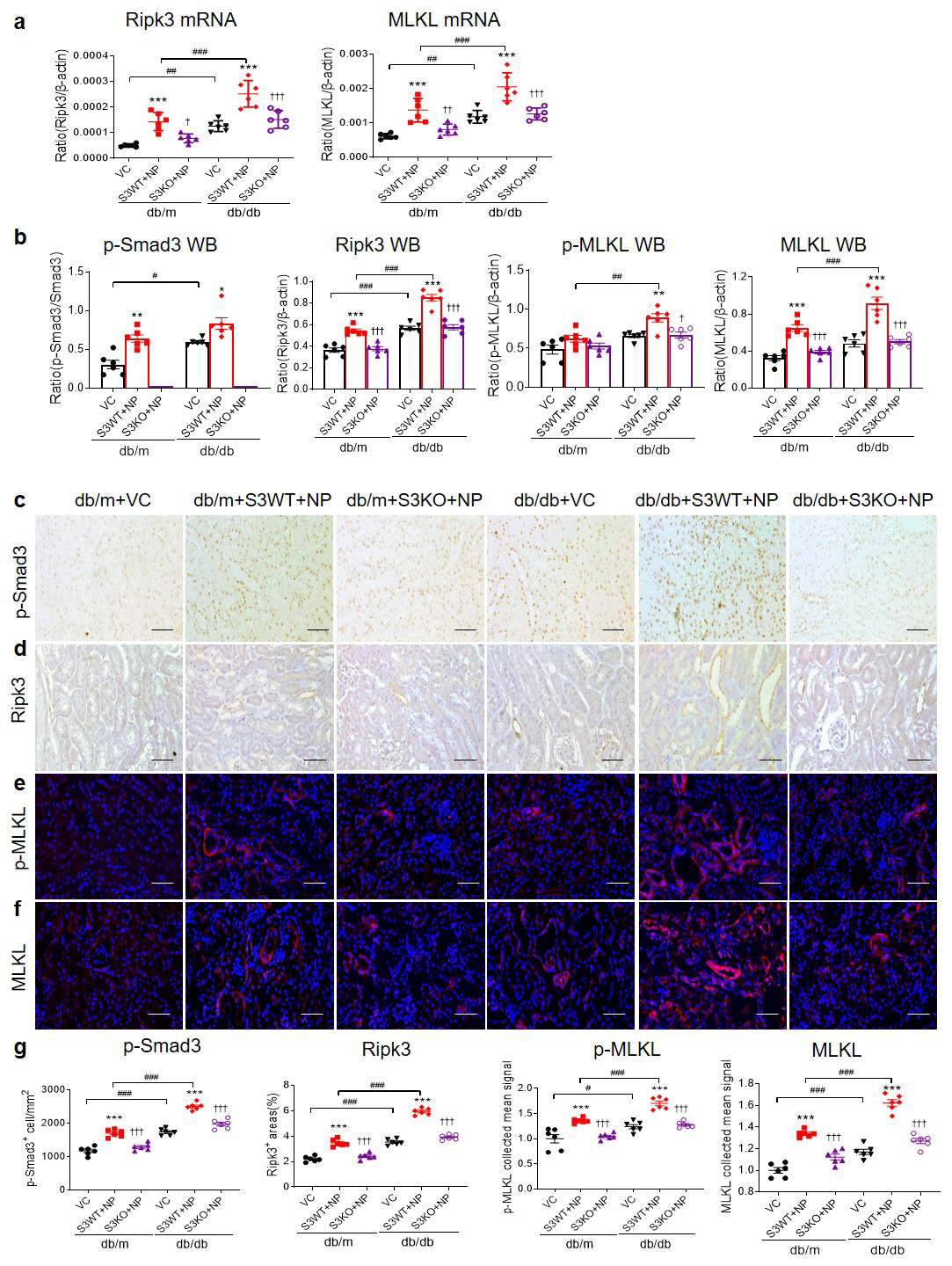


**Supplementary Figure S8. Deletion of Smad3 protects against SARS-CoV-2 N protein-induced AKI via the Smad3-Ripk3/MLKL necroptosis pathway.** **a.** Real-time PCR for Ripk3 and MLKL mRNA expression; **b.** Quantitative analysis of p-Smad3, Ripk3, p-MLKL and MLKL expression by western blot analysis; **c, d.** Immunohistochemistry for Smad3 activation (p-Smad3) and Ripk3 expression; **e, f.** Immunofluorescence for p-MLKL and MLKL levels (red); **g.** Semi-quantitative analysis for p-Smad3, Ripk3, p-MLKL, and MLKL expression. Note that compared to the db/m mice, overexpression of SARS-CoV-2 N protein enhances Smad3-Ripk3/MLKL signaling in db/db mice at the age of 16 weeks, which is abrogated by deleting Smad3. Each dot represents one mouse, and the data represents as the mean ± SEM for groups of 6 mice. **P* < 0.05, ***P* < 0.01, ****P* < 0.001 vs. VC group; ^†^*P* < 0.05, ^††^*P* < 0.01, ^†††^*P* < 0.001 vs. S3WT+NP group; ^#^*P* < 0.05, ^##^*P* < 0.01, ^###^*P* < 0.001 as indicated. Scale bar=50 μm.


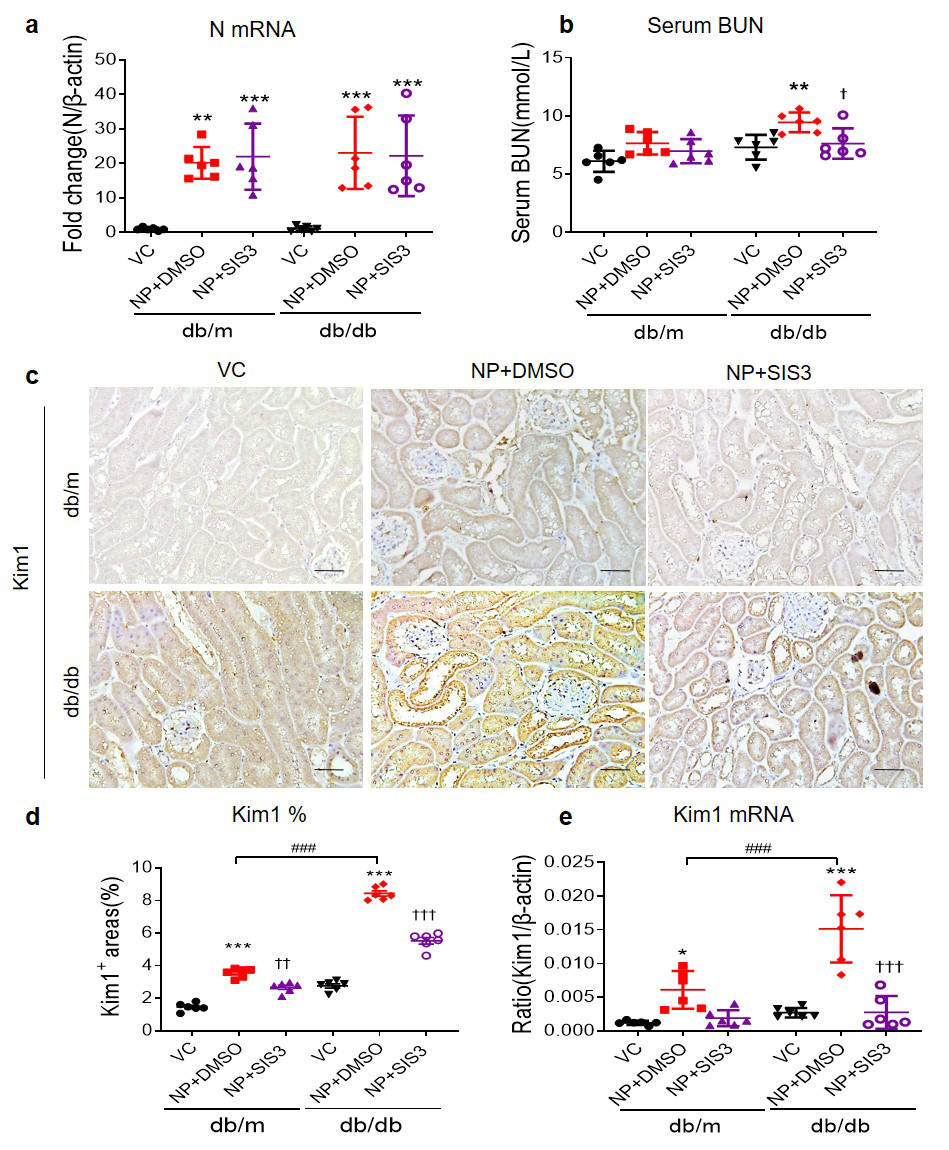
**Supplementary Figure S9. Treatment with SIS3 protects against SARS-CoV-2 N protein (NP)-induced AKI in db/m and db/db mice at the age of 16 weeks. a.** Quantitative real-time PCR for SARS-CoV-2 N mRNA expression; **b.** Serum levels of BUN; **c.** Immunohistochemistry for renal Kim1 expression; **d.** Semi-quantitative analysis of Kim1 expression; **e.** Quantitative real-time PCR for Kim1 mRNA expression. Note that compared to the db/m mice, overexpression of SARS-CoV-2 N protein (NP) induces AKI in db/db mice by significantly increasing serum levels of BUN and kim-1 expression, which is largely attenuated by treatment with SIS3 (10 mg/kg/day). Each dot represents one mouse, and the data represents as the mean ± SEM for groups of 6 mice. VC, empty vector control. **P* < 0.05, ***P* < 0.01, ****P* < 0.001 vs. VC group; ^†^*P* < 0.05, ^††^*P* < 0.01, ^†††^*P* < 0.001 vs. NP+DMSO group; ^###^*P* < 0.001 as indicated. Scale bar=50 μm.


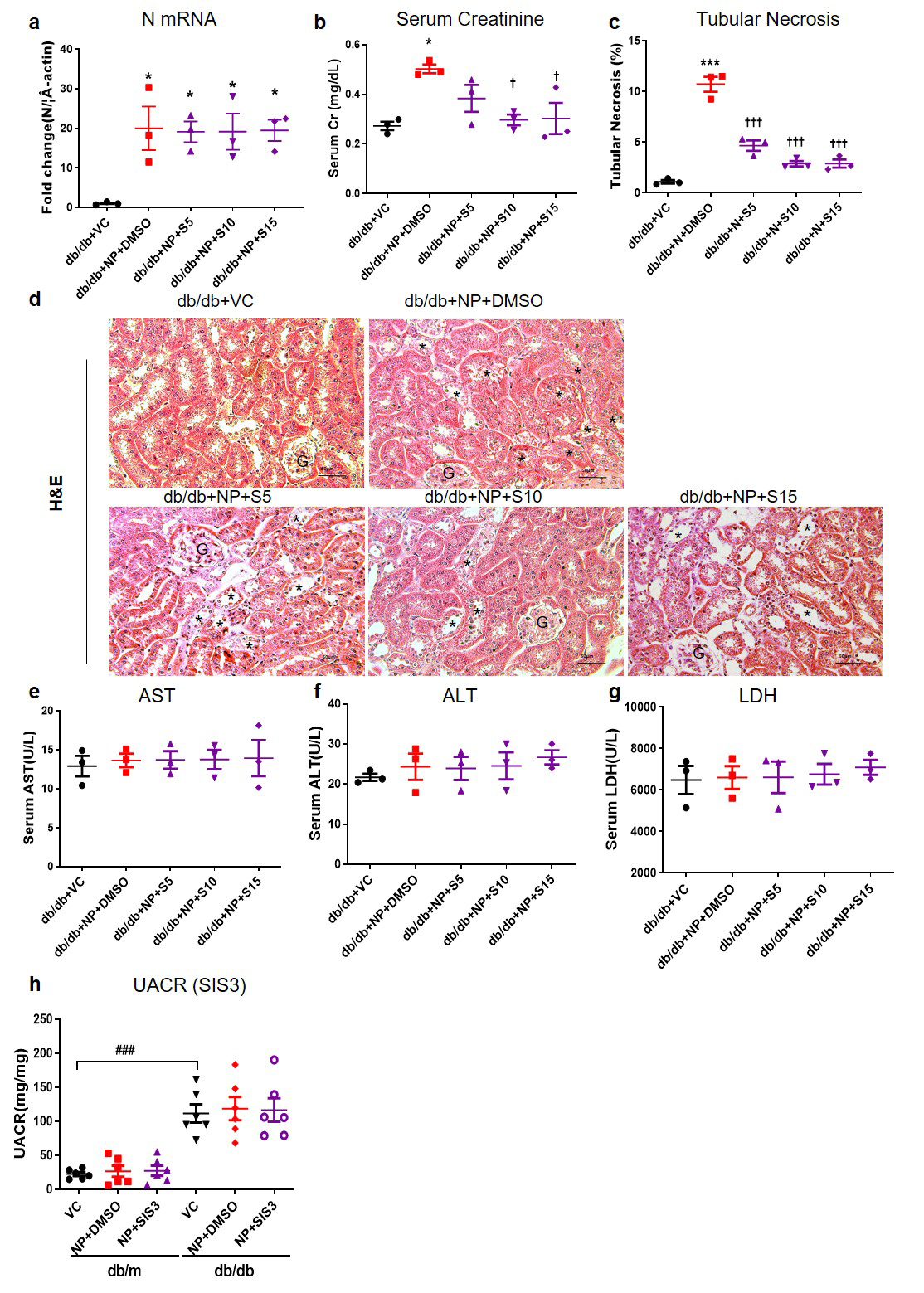


**Supplementary Figure S10. SIS3 treatment dose-dependently inhibits SARS-CoV-2 N–induced AKI in 16-week-old db/db mice without systemic toxicity.** Mice were injected with i.p. DMSO or SIS3 at dosages of 5, 10 or 15 mg/kg body weight daily from day 0 before SARS-CoV-2 N gene plasmid transfer until being sacrificed on day 2. **A.** SARS-CoV-2 N mRNA expression; **b.** Serum levels of creatinine; **c.** Semi-quantitative analysis of tubular necrosis; **d.** H&E- staining for detecting tubular necrosis (*); **e-g.** Serum levels of AST, ALT and LDH；**h.** Urinary albumin-to-creatinine ratio (UACR). Note that although db/db mice at the age of 16 weeks developed DKD with significantly increased UACR, it is not altered by SIS3, suggesting that a 3-day treatment with SIS3 do inhibit underlying DKD. Each dot represents one mouse and data are the mean ± SEM per group. *P < 0.05, *** p<0.001 vs. db/db+VC group, ^†^P < 0.05, ^†††^P < 0.001 vs. db/db+NP+DMSO group, ^###^P < 0.001 as indicated. G, glomerulus; Scale bar=50 μm.


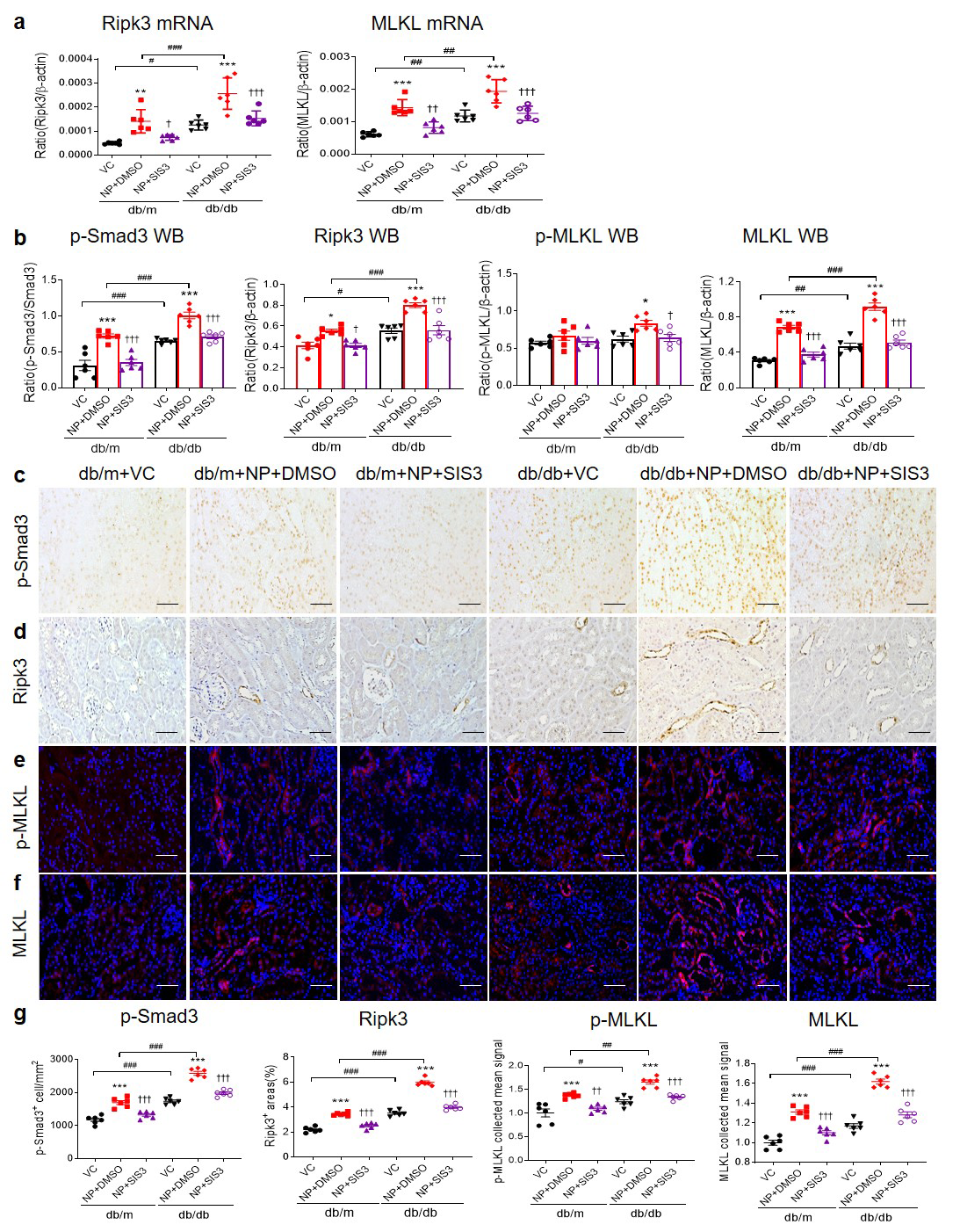


**Supplementary Figure S11. Treatment with SIS3 blocks SARS-CoV-2 N protein-induced Smad3- Ripk3/MLKL necroptosis pathway in db/m and db/db mice at the age of 16 weeks. a.** Quantitative real-time PCR for Ripk3 and MLKL mRNA expression; **b.** Quantitative analysis of p-Smad3, Ripk3, p-MLKL and MLKL expression by western blot analysis; **c, d.** Immunohistochemistry for Smad3 activation (p-Smad3) and Ripk3 expression; **e, f.** Immunofluorescence for p-MLKL and MLKL levels (red); **g.** Semi-quantitative analysis for p-Smad3, Ripk3, p-MLKL, and MLKL expression. Note that compared to the db/m mice, overexpression of SARS-CoV-2 N protein largely enhances Smad3- Ripk3-MLKL necroptosis pathway in db/db mice, which is blocked by treatment with SIS3 (10 mg/kg/day). Each dot represents one mouse, and the data represents as the mean ± SEM for groups of 6 mice. **P* < 0.05, ***P* < 0.01, ****P* < 0.001 vs. VC group; ^†^*P* < 0.05, ^††^*P* < 0.01, ^†††^*P* < 0.001 vs. NP+DMSO group; ^#^*P* < 0.05, ^##^*P* < 0.01, ^###^*P* < 0.001 as indicated. Scale bar=50 μm.

**
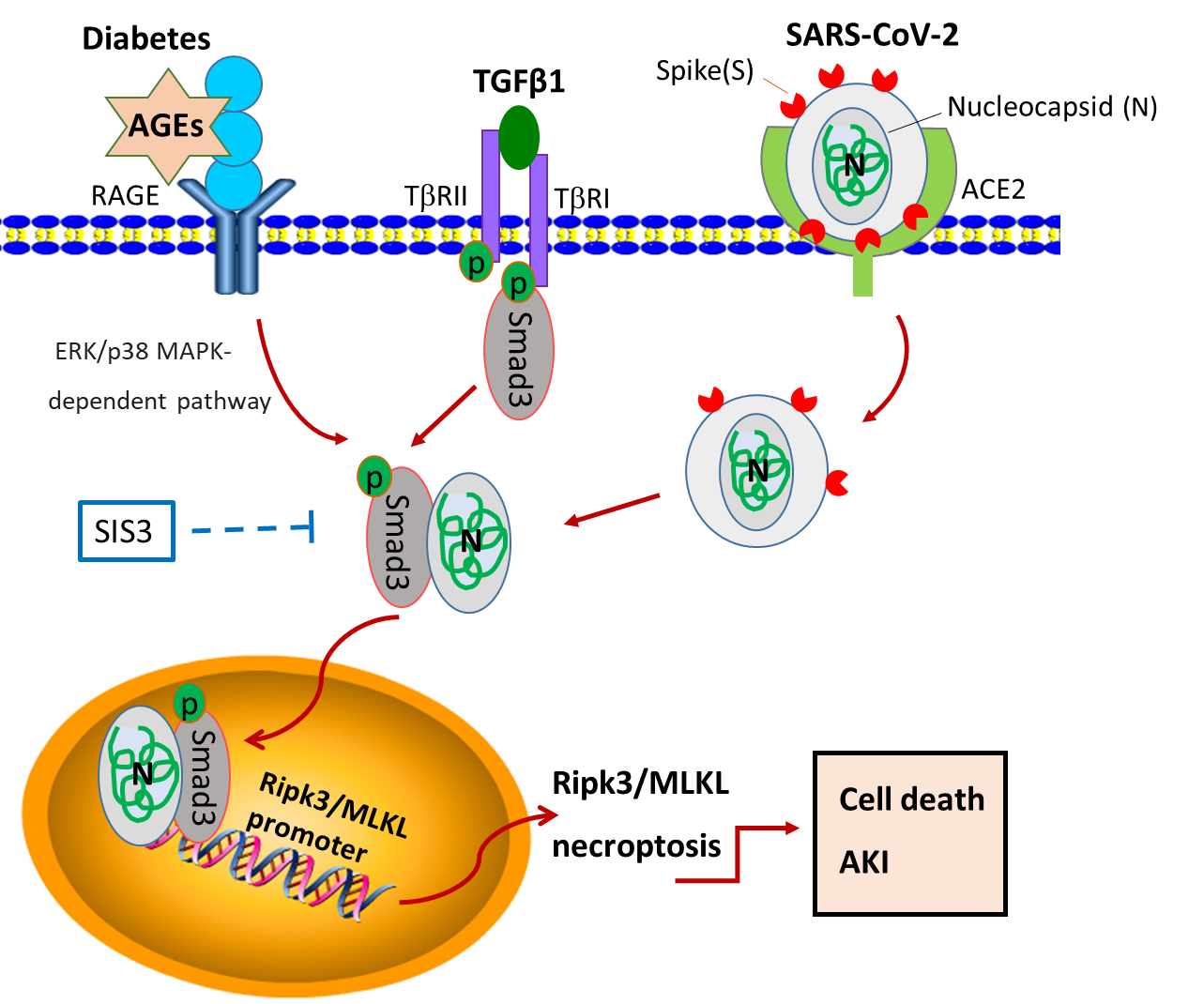
**

**Supplementary Figure S12. SARS-CoV-2 N Protein induces AKI in diabetic db/db mice via the Smad3- Ripk3/MLKL necroptosis pathway.**
